# Editorial: Brain and Cognition for Addiction Medicine: From Prevention to Recovery

**DOI:** 10.3389/fpsyt.2020.590030

**Published:** 2020-11-26

**Authors:** Hamed Ekhtiari, Antonio Verdejo-Garcia, Scott J. Moeller, Mehran Zare-Bidoky, Alexander Mario Baldacchino, Martin Paulus

**Affiliations:** ^1^Laureate Institute for Brain Research, Tulsa, OK, United States; ^2^Turner Institute for Brain and Mental Health, Monash University, Melbourne, VIC, Australia; ^3^Department of Psychiatry, Stony Brook Medicine, Stony Brook, NY, United States; ^4^School of Medicine, Shahid Sadoughi University of Medical Sciences, Yazd, Iran; ^5^Department of Psychiatry, University of St Andrews, St Andrews, United Kingdom

**Keywords:** neuroscience, addiction, neuroimaging, biomarker, brain stimulation, treatment, cognitive

In 2018, 269 million people around the world had used drugs, and over 35 million were suffering from substance use disorders (SUDs) (1). However, there is a serious limitation in the available treatments for SUDs that are effective in the long term (2–4). A question frequently raised by addiction medicine practitioners around the world is how recent advancements in different fields of brain and cognition studies—from molecular to cognitive neuroscience—can help them improve their daily practice for prevention, treatment, and rehabilitation of SUDs.

There is a growing body of evidence on neurocognitive alterations that contribute to developing a SUD and to hampering recovery, alongside a plethora of social and environmental factors (5, 6). However, there is a lack of neurocognitive markers and related outcome measures that are sufficiently sensitive and specific to addiction mechanisms, engaged by interventions, repeatable, and indicative of disorder progression and recovery. There is preliminary, but promising evidence for different neural and cognitive markers measured with brain mapping and cognitive assessments that (1) engage key mechanisms of addiction (incentive salience, negative emotionality, and cognitive control), (2) predict reduction of drug use (the gold standard for treatment outcomes), and (3) detect acute and chronic responses to interventions with therapeutic potential (7). However, none of these neurocognitive markers have yet approached formal qualification paths [e.g., Biomarker Qualification Program (BQP) of the FDA] or are being widely used in daily clinical practice. Some of the reasons that none of these markers are playing a formal role as a qualified biomarker in addiction prevention or treatment is because they lack methodological harmony, publicly available tools and normative databases, and strong replication and reliability/validity data.

Indeed, although there is a significant body of evidence from brain and cognition studies about SUDs, the impact of this evidence in the daily practice of addiction medicine is minimal and yet to be established. As part of our leadership roles in the *Neuroscience Interest Group* of the *International Society for Addiction Medicine* (ISAM-NIG), we believe that we need an orchestrated international effort to bring pieces of basic and clinical evidence together to develop a roadmap from bench to bedside and policy. We also need consensus and guidelines on how to translate currently available evidence to different dimensions of clinical practice, ranging from prevention to recovery.

In this cross-listed Research Topic in *Frontiers in Psychiatry* and *Frontiers in Human Neuroscience*, our overall goal was to invite researchers to provide evidence that can help bridge the gap between the neuroscientific knowledge of SUDs and its pragmatic use in routine clinical practice. In this successful Research Topic, we published 30 articles (17 original research articles, nine reviews, one systematic review, two mini-reviews, and one brief research report), from 146 authors from 13 countries that overall elicited 86,787 views at the time of submission of this editorial. Contributors to our Research Topic mainly sought to provide evidence on susceptibility/risk, diagnostic, predictive, and treatment monitoring evidence for different neural and cognitive markers. We also received articles providing evidence for different mechanistic-informed interventions (two cognitive/behavioral, one pharmacologic, and two brain stimulation interventions) that effectively engaged these markers. These markers spanned across molecular and biological assessments, genetics, different imaging techniques, cognitive assessments etc.

In this e-book, we (Verdejo-Garcia et al.) wrote a consensus paper with a group of ISAM-NIG members about strategies and suggestions to apply the neuroscientific knowledge of addiction medicine into daily practice which has shaped the scope of this Research Topic. In the following sections, we present select highlights of the contributions which we hope will convey a sense of how neuroscience can help increase the understanding of underlying mechanisms of SUDs and how it can inform the development of more impactful interventions.

## Evidence for Susceptibility/Risk Markers

A susceptibility/risk marker in addiction medicine can estimate how likely it is for someone to develop SUDs in the future. Burns et al. in their review discuss how molecular imaging shows that genetics can increase proneness to opioid use disorder and how these inter-individual differences in opioid and dopamine systems underlie the person's reward, cognition, and stress pathways leading to heightened risk of being an opioid user in the future. Among other contributions to this Research Topic, Abram et al. investigated undergraduate university students with a foraging task to assess their ability to associate reward pursuit and reward valuation. They found that in people with more externalizing traits, which confer risk for SUDs, pursuit and valuation were less related. Rose et al. propose distinctive pathways that may increase liability for developing SUDs. The authors discuss how addressing neural mechanisms that differentially characterize these pathways can inform preventive strategies, treatment development, and long-term outcomes. Thus, this e-book brings together promising results on how genetics can predict the level of cognitive functioning and how deficits or delays in specific cognitive dimensions might predict risk to developing SUDs. However, there remain several outstanding questions on the percent variance in this susceptibility/risk for developing a SUD that can be explained by cognitive and neural markers. Supporting evidence with validated cognitive and neuroimaging assessments will be needed on how these susceptibility/risk markers can be used in real world contexts to strengthen neural substrates and circuits of cognitive functioning in individuals at high risk of using preventive strategies/interventions to decrease the incidence of new cases with SUDs.

## Evidence For Diagnostic/Severity Markers

A diagnostic marker is used to identify subjects with SUDs. In the current Research Topic, researchers aimed to investigate how cognitive functions and imaging results differ between people with and without SUDs, and they report these differences among people with SUDs to illustrate how they are associated with other markers. Noorbakhsh et al. in a cohort study of 3,826 students from grades seven to eleven, found that among female students, working memory functioning, assessed by a neuropsychological test battery, was more negatively affected by the amount of cannabis use. The cause/risk/effect nature of these cognitive markers in relationship to SUD has yet to be explored. Tolomeo et al. showed that people with an opioid use disorder who received either methadone or buprenorphine treatment, have impaired visuospatial memory but those who are abstinent for a period of time do not. The authors also report that the impairment in visuospatial memory is correlated with higher mood and anxiety symptom severity scores. In a study conducted by Deldar et al. it was shown that abstinent methamphetamine users, in comparison with a control group, had lower reaction time in the Sternberg task when viewing drug-related stimuli. Schroder et al., in an ERP working memory task, found that hazardous alcohol drinkers have larger amplitude than light drinkers, mainly around P300 and P600 EEG components, which might be considered a diagnostic factor for risk of developing an alcohol use disorder. Sharman et al. found that two different subtypes of gamblers have different neuropsychosocial problems assessed by decision-making tasks and mental health indices; the authors suggest that treatment providers take these differences into consideration. Albein-Urios et al. evaluated psychological and cognitive problems in cocaine users and found that dysfunctional personality beliefs are correlated with poorer emotion recognition. Roberts et al., using a sample of daily smokers performing a Go/No-Go task after usual smoking and after a period of abstinence, found that during abstinence, smokers have faster information accumulation (accretion) with a lower threshold for prior information before execution (caution). Chen et al. showed that during an Implicit Association Test, people with an internet addiction, compared to controls, show increased activation in the occipital lobe measured by EEG. Jansen et al. (a) reported an fMRI study during an emotion regulation task and found that, although people with alcohol use disorder show no deficiencies in emotion processing compared to healthy people, they have reduced activation in the posterior insula, precuneus, operculum, and superior temporal gyrus when watching positive/negative cues. They also found that higher craving at baseline is associated with less reduced activation when viewing alcohol cues. Smallwood et al., in an fMRI study using structural equation modeling found that chronic pain and opioid use disorder have overlapping neural pathways. Common neural mechanisms and shared markers between chronic pain and opioid use disorder could inform future assessment and intervention studies. Coppens et al. in their review, summarize the role of inflammatory markers in cognition among people with alcohol use disorder; they detail how inflammation affects cognitive function and in turn how alcohol use impacts the inflammation. In conclusion, they suggest that inflammation may be a target in the treatment of alcohol use disorder.

Diagnosis of SUD is currently based on self-reports of use disorder signs and symptoms during structured clinical interviews; toxicology measures for presence of the drug or its metabolites in the human body are often used to corroborate use. The neurocognitive diagnostic/severity markers that are investigated in this Research Topic, along with thousands more annual publications in the field of addiction neuroscience, attempt to uncover sensitive, valid, and objective measures of mechanistic pathways specific to SUD to accurately assess SUD and its severity, ultimately leading to therapeutic intervention. Given the heterogeneity of deficits among people with SUDs, these diagnostic/severity markers might also be helpful to inform therapeutic interventions optimized for different subgroups within people with SUD. There is still a long road ahead to achieve this ambitious but vital goal.

## Evidence for Predictive/Prognostic Markers

Predictive markers estimate how likely it is that an individual with SUD would benefit from a certain treatment. Prognostic markers evaluate overall likelihood of recovery in the long term. Kearny-Ramos et al., in a single-blinded active sham-controlled crossover study, to evaluate the effect of medial prefrontal cortex (mPFC) using repetitive transcranial magnetic stimulation (rTMS) on drug cue-reactivity, found that lower striatal network activation at baseline predicts a higher change in this network in the participants after the act compared to sham. Destoop et al. conducted a systematic review and concluded that anhedonia associated with SUDs negatively affects the success of treatment in long-term.

As reported in this Research Topic, there are hopes that different neural and cognitive markers can help determine the likelihood of the person responding to a specific treatment or recovery/abstinence in general. Ultimately, these markers should inform clinical decision making to optimize the preventive/therapeutic intervention at the individual level.

## Evidence for Monitoring Markers

Monitoring markers are used with the goal of evaluating the effectiveness of a treatment by assessing whether that treatment can change a mechanistic impairment in a person with SUDs. Stewart et al. reviewed opioid use disorder in a three-stage brain model with negative reinforcement processes, binge/intoxication processes, and preoccupation/anticipation processes. They continue by evaluating neuroimaging studies on opioid use disorder monitoring the effects of different interventions in both cross-sectional and longitudinal settings and discussing their limitations and strengths. They conclude with recommendations for future neuroimaging research of opioid use disorder. Vonmoos et al., in a cohort study on chronic cocaine users, assessed socio-cognitive deficits and cluster B personality disorder symptoms, and showed that they are negatively correlated with the change in the amount of substance use following 1 year after baseline assessments. There is still no FDA approval for any neural or cognitive marker to be used as a proxy measure for substance use recovery in clinical trials. However, studies in this area may open doors for novel monitoring markers which serve as key dependent variables in intervention development for addiction medicine.

## Evidence for Mechanism-Informed Interventions

The ultimate goal of all types of markers introduced above is to first target and accurately measure a mechanistic deficit in people susceptible to or who suffer from SUDs, which then informs therapeutic interventions to modulate the deficit. The feedback loop between the mechanistic markers and interventions should pragmatically lead to new and better tailored interventions (8). In this Research Topic, we published different sample interventional studies trying to contribute to this marker/intervention feedback loop. These mechanism-informed interventions could be categorized into cognitive/behavioral, pharmacologic, and brain stimulation interventions.

### Cognitive/Behavioral Interventions

Halcomb et al. review methods to measure negative urgency in cross-species translational studies, how negative urgency can inform treatment development, and provide some suggestions for the future direction of the field. Contributing to this Research Topic, Grodin et al., in an fMRI study of heavy alcohol users, assessed the motivation to change after one session of brief drinking intervention. They found that the individuals who received real intervention compared to a sham intervention, had higher scores in the importance to change, and this was associated with higher activation in the precuneus, posterior cingulate, and insula during fMRI alcohol cue-reactivity task. Costa et al. reviewed the role of physical exercise as an adjuvant to routine substance use treatment. The beneficial effect of exercise may be attributable to improving executive function. Kouimtsidis et al. discuss how pre-rehabilitation plays a significant role in successful alcohol detoxification. In a clinical trial with neurocardiac modulation, Bates et al. showed that cardiac resonance paced breathing can alter alcohol cue reactivity in persons with an alcohol use disorder. The active intervention group compared to the sham group showed lower activation to alcohol cues in visual areas, and increased activation in self-control, directed cognition, and brain-body integration areas. Behavioral manipulation of the baroreflex mechanism extends neuroscience-informed addiction intervention approaches to include modulation of bi-directional signaling between the brain and the cardiovascular system.

### Pharmacological Interventions

Joseph et al. reported the results of a trial using a graph-theory functional connectivity analysis and machine learning as a monitoring marker among people with cocaine use disorders to assess the effect of oxytocin on resting-state fMRI. The authors found that oxytocin compared to a placebo increases the connectivity between salience nodes and default mode network nodes differently among women and men, and that childhood trauma and years of cocaine use modulated the effect. Chye et al. first discuss the role of the endocannabinoid system in SUDs and then review the role of cannabidiol on SUDs treatment. This evidence leads to a discussion on potential pharmacological interventions targeting the endocannabinoid system in people with SUD. Butler and Le Foll in their review cover various pharmacotherapies used to treat SUD and to determine how they affect the executive functions of the participants, why there are mixed results, and how to move forward with using both pharmacological and non-pharmacological therapies to enhance cognitive functioning.

### Brain Stimulation Interventions

Jansen et al. (b) assessed the effect of right dlPFC-rTMS on emotional processing, reappraisal and craving, and their neural correlates by fMRI during an emotion reappraisal task among people with alcohol use disorder. They found that rTMS compared to a sham reduces dlPFC activation and also modulates self-reported experienced emotions. However, they were unable to find any change in the craving levels, or on reappraisal related brain function.

Altogether, the articles included in this Research Topic on mechanism-informed interventions, along with trials using monitoring markers, illustrate the breadth and depth of international efforts to enhance the feedback loop between markers and interventions in addiction medicine. We endeavor to coordinate and harmonize these efforts as a necessary next step to consolidate research advances and to foster pragmatic clinical translation.

We request funding agencies around the world to support studies that aim to generate datasets that enable researchers to rigorously examine the reliability and validity of neural and cognitive markers, with a goal to establish performance of these markers sufficient to meet formal biomarker qualification standards, similar to that offered by the FDA (9). Our shared long-term goal within the community of addiction neuroscientists is to establish publicly available neural and cognitive markers and their tools, which can be used broadly by multiple investigators (10, 11). This approach will accelerate intervention development and provide outcome measures in RCTs in research settings that can ultimately be used to predict treatment response, inform personalized treatment selection, and monitor treatment efficacy in daily clinical practice.

To reach this goal, we propose the following as initial steps. (1) We need to determine the relationship between true and observed effect sizes with proposed neural and cognitive markers using test-retest reliability measures like intraclass correlation coefficient (ICC). This is a critical need that has not yet received enough attention. (2) We need to determine the validity (risk/susceptibility, diagnostic, predictive, and treatment monitoring) of proposed neural and cognitive markers as biomarkers. (3) We need to repeat Steps 1 and 2, searching for the best set of derived multivariate measures and their pre-registered analysis pipelines in different subjective, physiological, immunological, neural, cognitive, and behavioral markers. Using machine learning methods with proper linear and non-linear models and cross-validation will increase confidence for reasonable replicability (12). (4) Then ultimately, we need to compile, collect, and aggregate the best measures with optimum reliability and multi-dimensional validity based on the standards for biomarkers to inform future mechanism-based intervention development. These resources of tasks/tests of known reliability/validity should be publicly available in repositories like Github or open science framework (OSF) platforms (13).

We further assert that there is a need for methodological checklists to harmonize the parameter space in the field and to promote transparency. As an example, we are working on a new methodological checklist we have recently put forward within the ENIGMA addiction cue reactivity initiative (ACRI) to promote harmonization and open sourcing within the community of labs using fMRI drug cue reactivity as a potential biomarker (14). We encourage addiction neuroscientists to work on similar checklists for other core phenotypes. The successful completion of the proposed pathway in this editorial has the potential to yield a set of brain-based biomarkers for SUDs that can be used in research and practice in addiction medicine.

## Author Contributions

HE and MZ-B have prepared the initial draft of the editorial. All authors have contributed to make the final draft of the editorial. All authors have agreed on the final draft of the editorial.

## Conflict of Interest

The authors declare that the research was conducted in the absence of any commercial or financial relationships that could be construed as a potential conflict of interest.
